# The impact of gender differences in teachers’ teaching practices and attitudes on students’ math and science achievement in Saudi Arabia: Evidence from TIMSS 2019 data

**DOI:** 10.3389/fpsyg.2023.1066843

**Published:** 2023-02-23

**Authors:** Ghaleb Hamad Alnahdi, Susanne Schwab

**Affiliations:** ^1^Department of Special Education, College of Education, Prince Sattam Bin Abdulaziz University, Al-Kharj, Saudi Arabia; ^2^Center for Teacher Education, University of Vienna, Vienna, Austria; ^3^Research Group Optentia, North-West University, Vanderbijlpark, South Africa

**Keywords:** gender differences, math, science, TIMMS, attitudes, teaching practices, Saudi Arabia

## Abstract

Previous results from large-scale studies often identified a gender gap in achievement outcomes between girls and boys. This study aims to investigate the existence of a relationship between students’ grades and gender differences in practices and attitudes among teachers. Data from the Saudi Arabian TIMSS 2019 were analyzed for this study. The dataset contains data from 10,817 4th grade students (5,447 girls and 5,370 boys) who took math and science as well as data from their 437 teachers (around 50% were female teachers). The results showed that teachers’ teaching practices were positively associated with students’ scores in math and science. In addition, teachers’ attitudes toward teaching were positively associated with students’ scores. Female teachers held more positive attitudes toward teaching and were rated more positively in terms of practices by their students.

**Highlights:**

– Teacher practices were positively associated with students’ achievement.

– Teachers’ attitudes were positively associated with students’ achievement.

– Female teachers hold more positive attitudes toward teaching.

– There are differences in teaching style and practices based on gender.

## Introduction

Within large international achievement assessments such as the Program for International Student Assessment (PISA) or Trends in International Mathematics and Science Study (TIMSS), gender differences in student achievement have been widely studied (see e.g., Reilly et al., 2019; Eriksson et al., 2020). For instance, Voyer and Voyer (2014) pointed out in their meta-analysis that girls currently achieve higher scholastic achievement than boys. Furthermore, the authors also stated that the size of the gender gap in scholastic achievement varies between countries.

**Figure 1:**
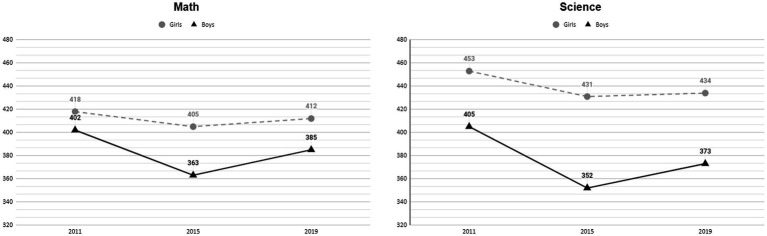
Average mathematics and science achievement across years by gender.

The TIMSS started in 1995 to examine trends in mathematics and science achievement across different countries every 4 years. The TIMSS is conducted among students in the fourth and eighth grades (Mullis and Martin, 2017; Fishbein et al., 2021). The Kingdom of Saudi Arabia participated in the last five versions of the TIMSS (2003, 2007, 2011, 2015, and 2019) (Shahada and Alqramiti, 2016). Participating in these tests is one of the indicators that Saudi Arabia has recently increased efforts to monitor students’ performance and progress in a context with their peers in developed countries. These data give officials in Saudi Arabia a better picture of student progress and school outcomes. Saudi Arabia is one of only four countries in which girls outperform boys in the same country in fourth-grade math (Mullis et al., 2020) and one of 18 countries in which girls outperform boys in fourth grade science (Mullis et al., 2020) (see Figure 1). This result is also similar to the results in 2011 and 2015, where girls outperformed boys in Saudi Arabia (Mullis et al., 2020). After the results of the TIMSS 2019 were released, we found that the results of students in fourth grade in Saudi Arabia were low in comparison with other countries. The average Math score in Saudi Arabia was 398, and the average score for Science was 402, which were both below the TIMSS scale center points (Mullis et al., 2020). For the girls, the average scores were 414 and 434 for math and science, respectively, while for the boys it was 385 and 373. This discrepancy and low scores from the boys’ side affect the total average. In this context, Almulhim (2021) indicated the importance of studying gender differences in various evaluation studies.

In this vein, especially in countries such as Saudi Arabia, where boys and girls are taught in separated schools in all different stages of education from the elementary stage to the end of postgraduate studies in university, these gender differences are of high interest.

However, while students’ gender was taken into account extensively, there is a lack of literature focusing on teachers’ gender and possible effects on students. Moreover, according to the subject, gender gaps in science and mathematics might be of special interest, as females in general are underrepresented in such topics (OECD, 2011).

### Teacher variables linked with students’ achievement

Over the last several decades, linking teacher variables with students’ outcomes has increased. Empirically, teachers’ key role in influencing students’ scholastic outcomes can be underpinned by residual variance of students’ achievement at the class level (see e.g., Ingvarson and Rowe, 2008). For instance, previous literature has reported a positive relation between years of teaching experience and student outcomes (e.g., Wayne and Youngs, 2003; for an overview, see also Burroughs et al., 2019). However, some studies failed to confirm this effect (e.g., Blömeke et al., 2016; Gustafsson and Nilsen, 2016). In addition, teachers’ major might be positively linked with students’ outcomes, as teachers’ knowledge was shown to be a significant predictor of students’ achievement. For instance, the results from Gess-Newsome et al. (2019) indicated that academic content knowledge is significant for students’ achievement. In the context of Saudi Arabia, a study by Dodeen et al. (2012) compared Saudi and Taiwanese teachers’ qualifications, practices, and perceptions in relation to students’ achievement in math in TIMSS 2007. The results indicated somewhat lacking qualifications of Saudi Arabian teachers to teach mathematics. Furthermore, their results indicated a link between teachers’ qualification and teaching practices and students’ mathematics achievement. A further variable associated with students’ outcomes was teachers’ attitudes toward teaching and learning itself (see, e.g., Akinfe et al., 2012). A higher level of teachers’ job satisfaction also has positive effects on students’ outcomes (see e.g., Skaalvik and Skaalvik, 2015). Furthermore, teaching styles and practices affect students’ outcomes. Teaching styles that encourage students to be emotionally, cognitive or physically involved impact their learning processes. For instance, the results from Clanet (2010) indicated a link between students’ achievement and teaching activities that supported and encouraged students. Furthermore, previous literature has indicated that teaching styles that are more focused on student-centered learning processes are positively linked with students’ achievement (see, e.g., Al-Agili et al., 2012; Clements et al., 2013). Furthermore, the results from Ngware et al. (2014) indicated that teaching styles are associated with students’ learning outcomes. However, within the study of Van der Zanden et al. (2021), no evidence was found for a relation between teaching practices and students’ academic achievement.

In Saudi Arabia, some researchers have highlighted the impact of teachers on students’ performance on internal national tests. For example, Almulhim (2021) discussed the impact of teachers on the low results of students in Saudi Arabia on international tests. In addition, discussed that although the mathematics and science curricula are similar to what is offered to students in some developed countries, teachers’ scores are low in the results of their tests of teaching competencies. He stated that “how do we expect a student to excel and this is the level of his or her teacher,” p. 18. Another possible way to improve students’ performances is to increase the number of lessons in mathematics and science in Saudi schools (ALshuweil, 2021).

### Saudi Arabian teachers’ gender gaps in teaching attitudes, teaching style and teaching practices

The school system in Saudi Arabia includes single-sex classrooms taught by teachers of the same gender (Hamdan, 2005; Haroun et al., 2016). Although male and female teachers work in separate schools, there are many similarities between them. For example, teacher training curricula in universities are identical for both genders. This is ensured as teacher training is prepared by the same academic departments that are administered in a joint way between faculty members of both sexes. Both female and male teacher training students have to pass professional licensing tests for teachers who are administered annually by the Education and Training Evaluation Commission (ETEC) in Saudi Arabia. In addition, the procedures for appointing male and female teachers in the Ministry of Education and appointing them to the same profession levels with identical salaries were based on the qualifications.

Within the context of single-sex classrooms in Saudi Arabia, it will be analyzed whether gender gaps in teachers can partly explain gender differences in students’ achievement. To date, only limited results are available about gender differences between male and female teachers in Saudi Arabia. However, previous results from other countries and contexts indicated that teachers’ gender might matter. Chudgar and Sankar (2008) pointed out (for Indian teachers) that females (compared to males) tend to be more encouraging of the learning of all children. According to the literature review of Unterhalter et al. (2014), the employment of female teachers is associated with the enhancement of girls’ learning outcomes.

A study by Haroun et al. (2016) examined Saudi Arabian teachers’ gender differences in mathematical knowledge for teaching. Their results indicated that female teachers have a higher level of mathematical content knowledge than male teachers. According to teachers’ attitudes in the Saudi Arabian context, Almulla (2020) identified different patterns in male and female teachers’ motivation to be a teacher. For instance, the level of job satisfaction and motivation toward the teaching profession were higher among female teachers than among male teachers. Regarding gender differences in job satisfaction, studies from other countries have also indicated higher job satisfaction in female teachers. Toropova et al. (2020) used data from the TIMSS 2015 and showed that Swedish female teachers scored higher in job satisfaction than their male counterparts. Moreover, analyses of TIMMS 2015 data showed that there is a significant connection between students’ achievement and teachers’ gender (see Hastedt et al., 2021). Hastedt et al. (2021) analyzed data from TIMMS 2015 and stated that students who are taught by female teachers had higher achievements in mathematics and science. This teacher gender gap was found for both grades, 4^th^ and 8^th^ graders. Interestingly, the results show that boys succeeded more when taught by female teachers. For example, for 4^th^ graders in mathematics in 6 out of 52 education systems, boys benefited from having female teachers. Similar results were shown for 8^th^ graders in 11 out of 43 education systems. However, as Hastedt et al. (2021, p. 5) stated, “Saudi Arabia uses single-sex education. The relationship between the gender of the teacher and student achievement in the context of single-sex education warrants further research.”

## Current study

To study the variables that may have a role in this disparity between girls and boys, we analyzed TIMSS 2019 data from Saudi Arabia to examine the relationship of five factors related to teachers to verify the most related factors to the discrepancy in fourth grade students’ achievements in mathematics and science. These variables were teaching attitudes, teaching style, teaching practices, years of experience, and major.

Because of the existence of this clear and frequent discrepancy, the importance of this study came with the intention of seeking some understanding of some of the variables that may play a role in this disparity between the performance of girls and boys in the fourth grade.

Despite the fact that the average student performance is low compared to developed countries, this is in comparison at the international level, and there is a need for improvement in the performance of students. We should remember two main points. First, there is also a gap in the performance of male students that must be addressed to reach the level of female students in Saudi Arabia as a first step to take it to the next step to the improvement of students as whole compared to other countries. Second, with knowing that the main challenge that the average performance of students in Saudi Arabia is at levels lower than the general average of performances on the math and science tests, there is a discrepancy according to the gender of students and teachers and not only the gender of students. It is one of the countries that needs to be taken into account, when comparing the performance of male and female students, that this comparison involves comparing the output of female teachers with male teachers.

The school system is totally separated by gender. However, it is managed by the same ministry, so there are no differences in terms of regulations and laws that rule school weather boys or girls. Therefore, this study focuses on the clear differences between the two schools, which is the teacher’s gender, and attempts to understand whether female or male teachers’ practices, teaching style or attitudes are associated with students’ achievement. This study main assumption that teachers’ dispositions relate with their gender might influence students’ achievement in math/science.

Therefore, the main research question is:

Do differences in male and female teachers’ teaching styles, attitudes and teaching practices explain differences in students’ achievement?

## Sample and Instruments

The sample in this study was nearly split into two halves based on teachers’ and students’ gender. The distribution of students were boys (49.6%) and girls’ students (50.4%). This distribution was similar for teachers (n = 437) with 49.7% were male teachers and 50.3% were female teachers. Similar. Data were collected from 220 schools with an average of 49 students from each school. According to ETEC, the tests were administered in Saudi Arabia on 9–10 April 2019.

The instruments used in this study were part of the data collected for the TIMSS 2019. Five variables were the focus of this study as predictors. The data for these variables were collected according to the following details:

### Attitudes toward teaching

This questionnaire was answered by teachers and included six items to measure participants’ attitudes and feelings about being a teacher, such as “*I find my work full of meaning and purpose*,” “*I am enthusiastic about my job*” and “*I am proud of the work I do*.” A four-point Likert-type scale was used to answer these items, ranging from “*very often*” = 1, “*often*” =2, “*sometimes*” = 3 to “*never or almost never*” = 4. The Cronbach’s alpha for this scale was 0.809.

### Teaching style

This questionnaire was answered by teachers and included eight items to examine participants’ teaching style with items such as “*Encourage classroom discussions among students,” “Relate the lesson to students’ daily lives,” and “Link new content to students’ prior knowledge.”* A four-point Likert-type scale was used to answer these items, ranging from “*every or almost every lesson*” = 1, “*about half the lessons*” =2, “*some lessons*” = 3 to “*never*” = 4. The Cronbach’s alpha for this scale was 0.76.

### Teaching practices

This questionnaire was answered by students and included six items to examine how often teachers applied good teaching practices from students’ perspectives with items such as “*My teacher explains a topic again when we do not understand,” “My teacher has clear answers to my questions,” and “My teacher does a variety of things to help us learn.”* A four-point Likert-type scale was used to answer these items, ranging from “*agree a lot*” = 1, “*agree a little*” =2, “*disagree a little*” = 3 to “*disagree a lot*” = 4. The Cronbach’s alpha for this scale was 0.752 for math and 0.776 for science. For the three instruments, the Crobach’s alpha ranged from 0.754 to 0.809, and these values are good indicators of internal consistency (Taber, 2018) among items within each scale.

### Teaching years of experience

Years of experience ranged from a first year of working as a teacher to 36 years of experience.

### Teacher age

Less than 10% of the teachers were under 30 years of age, and 40% were between 30 and 39 years of age.

### Teachers major

Teachers were asked to specify whether they specialized in math (no = 0, yes = 1) and other items about science (no = 0, yes = 1).

## Results

We examined the predictability of five variables on students’ scores on math and science (see Table 1). The five predictors were teachers’ practices (students’ perspective), teaching style (teacher perspective), teachers’ attitudes toward teaching, teaching years of experience, and teacher major (whether teachers were specialized on the subject, yes/no). The multiple regression showed that teachers’ practices (students’ perspective), teaching style (teacher perspective) and teachers’ attitudes toward teaching were the three highest predictors of students’ achievement in both math and science. The model that included these five predictors was significant for both math (F5, 8,313 = 99.764, *p* < 0.001, *R*^2^ = 0.057) and science (F5, 8,062 = 146.804, *p* < 0.001, *R*^2^ = 0.083). The best predictor in both models was teachers’ practices (students’ perspective), with partial *R* = 0.180, which means that this variable alone could explain approximately 3.2% of the unique variation in students’ math scores. Similar results were obtained to predict science scores, where teachers’ practices (students’ perspective) obtained partial R = 0.206, which could mean that this variable could explain approximately 4.2% of the unique variation in students’ scores.

**Table 1:** Multiple regression coefficients.

DV^a^	*P*^b^	Unstandardized coefficients		Standardized coefficients	*t*	Partial^c^
		*B*	Std. Error	Beta		
Math	(Constant)	510.224	6.335		80.538***	
	Teachers’ practices	−30.810	1.843	−0.179	−16.717***	**−0.180**
	Teaching style	−20.801	2.444	−0.096	−8.510***	**−0.093**
	Teaching attitudes	−21.762	3.119	−0.078	−6.976***	**−0.076**
	Years of experience	0.363	0.131	0.030	2.772**	−0.030
	Teacher’s major Study\Mathematics	−4.280	2.183	−0.021	−1.961	
Science	(Constant)	555.758	6.900		80.542***	
	Teachers’ practices	−36.767	1.948	−0.203	−18.872***	**−0.206**
	Teaching style	−33.757	2.703	−0.141	−12.490***	**−0.138**
	Teaching attitudes	−25.151	3.476	−0.081	−7.236***	**−0.080**
	Years of experience	0.186	0.145	0.014	1.288	–
	Teacher’s major Study\Science	−4.955	2.410	−0.022	−2.056*	−0.023

*** significant at *p* < 0.001, ** significant at *p* < 0.01, * significant at *p* < 0.05. a, dependent variable; b, predictors; c, partial correlation; bold, top three predictors.

### Gender differences

Next, we found that the three variables related to teachers’ style, practices, and attitudes were positively associated with students’ achievement in math and science. A comparison was conducted between female and male teachers on these three variables to see if that could explain some of the variation in the gap between boys and girls. Table 2 shows that there were statistically significant mean differences on the four variables with higher levels on these variables for female teachers.

**Table 2:** Means, Cronbach’s alpha, and test of differences by gender.

	Female M (SD)	Male M (SD)	Mean differences	Alpha^a^
Teachers’ practices (students’ perspectives-math)	1.30 (0.47)	1.51 (0.65)	0.21**	0.752 (6^b^)
Teachers’ practices (students’ perspectives-science)	1.31 (0.51)	1.53 (0.66)	0.22**	0.776 (6^b^)
Teaching style (teachers’ perspective)	1.51 (0.39)	1.86 (0.46)	0.35**	0.761 (8^b^)
Teaching attitudes	1.15 (0.29)	1.31 (0.42)	0.16**	0.809 (5^b^)

^**^ANOVA *p* < 0.01. a, Cronbach’s alpha; b, number of items.

In addition, to make it easy to see some examples of the teachers practices items. For example, items such as “*Teacher explains good*” in Science were significantly associated with students’ achievement (*R* = 0.259) for both girls and boys. *“Teachers explains again*” as an item was significantly correlated (*R* = 0.199, and *R* = 0.20) with math and science, respectively, from the girls’ sample and from the boys’ sample (*R* = 0.20 and *R* = 0.22) with math and science, respectively (see Tables 3, 4). Regarding the importance of teachers’ attitudes toward teaching, the item “*Being a teacher inspires me*” is significantly correlated with achievement in math and science (*R* = 0.10 and 0.12, respectively) for the whole sample.

**Table 3:** Practices, style and attitudes correlations with math scores.

	Practices by math teacher^a^						Overall (practices)			Teaching attitudes	Teaching style
Gender	Clear expectations	Easy to understand	Clear answers	Explains good	Does a variety	Explains again					
Female	0.044**	0.088**	0.109**	0.152**	0.154**	0.199**	0.183**	0.045**	0.016
Male	−0.032*	0.108**	0.126**	0.160**	0.120**	0.200**	0.165**	0.122**	0.151**
All	0.018	0.118**	0.138**	0.168**	0.150**	0.212**	0.196**	0.120**	0.132**

*p < 0.05, **p < 0.01. a, teacher practices from students’ perspectives.

**Table 4:** Practices, style and attitudes correlations with science scores.

Gender	Practices by science teacher^a^						Overall (practices)	Teaching attitudes	Teaching style
	Clear expectations	Easy to understand	Clear answers	Explains good	Does a variety	Explains again			
Female	0.025	0.119**	0.162**	0.208**	0.193**	0.203**	0.216**	0.054**	0.034*
Male	−0.054**	0.118**	0.196**	0.243**	0.157**	0.124**	0.180**	0.113**	0.156**
All	0.000	0.149**	0.216**	0.259**	0.206**	0.189**	0.237**	0.144**	0.187**

*p < 0.05, **p<0.01. a, teacher practices from students’ perspectives.

To conclude, teachers’ practices, style and attitudes were significant predictors of students’ achievement in math and science. Female teachers on these three variables outperformed male teachers. This might explain some of the variation in students’ scores on math and science, which led to girls scoring significantly higher than boys.

## Discussion

This study is among the first to attempt to understand the effect of teacher gender on student achievement in mathematics and science, especially within the context of single-sex classes in Saudi Arabia. Most of the studies discussed the disparity in achievement according to the gender of the students without considering the teacher variables as possible predictors. The results of this study showed that the disparity in the performance of girls and boys might be a partial reflection of their teachers’ practices and attitudes, which were more positive among female teachers than among male teachers. This was evident from data reported by teachers themselves and data reported by students about their teachers’ practices.

The positive impact of having female teachers on students’ scores was not only found in Saudi Arabia. Hastedt et al. (2021) signaled the results of the TIMSS 2019 and confirmed that there are more education systems where there is a positive association between students’ achievement in science and mathematics and being taught by female teachers. The differences in the case of Saudi Arabia were that the impact of female teachers on girls was clear, but there was no clarity regarding whether female teacher would have a similar impact on boys due to the country’s same-sex education system. Internationally, the picture is still not clear, and some studies have found mixed results on the different gender relationships between teachers and students (see Cho, 2012; Spilt et al., 2012).

To understand the disparity between female and male teachers in these results, the findings could be examined from different perspectives. First, it is possible that inputs in female programs for teacher preparation are from the best groups of high school graduates because of the limited options in universities for female graduates in previous years, which have started to change. Therefore, the positive indicators from female teachers in this study sample could be explained by the limited options in universities for female students increasing the probability of having top students in education colleges. The case could be totally different for male graduates from high schools with a wide range of options of disciplines in universities in Saudi Arabia. It can be assumed that in the future, females who choose to be teachers will not be from the top groups as in previous years, due to their increasing job prospects in recent years. These points lead us to assert the importance of selecting top students to be teachers. This might lead to a question that needs to be asked: ‘*Is the teaching profession among the top attractive jobs in the country nowadays?’*. The short answer would be ‘*no*’. Therefore, the dilemma will continue regarding how to recruit top students into the teaching profession.

The results of this study show us that one of the important points that might help improve students’ achievement is to be taught by highly motivated teachers who are showing truly positive attitudes and using high-quality teaching practices. Future research would need to determine why these attributes are less common among male teachers. There is also a role for the Ministry of Education to play in this regard, as the MOE is the highest authority for both teacher preparation programs and teachers. Working on improving this issue would contribute to improving the level of students’ performance and increasing the average general scores of all students in Saudi Arabia. This step is also needed to achieve another goal, which is to seek to catch up with the high achieving countries in the international tests.

This study highlighted the importance of teachers’ attitudes and practices on students’ performance. This makes ignoring the attitudes of individuals during teacher selection difficult to understand, both at the level of preparation at the college level and by the main employer at the Ministry of Education (MOE).

## Educational implications

Different implications can be drawn from these study results. Improving the status of the teaching profession is a key element in the development of any education system. It is important to attract top students to choose teaching as their profession. This can be seen in some of the top countries in education systems based on students’ performance, such Finland, where only approximately 10% of applicants are accepted to be teachers in primary schools (Sahlberg, 2010). In this context, the MOE discusses that as on the main issued to improve the education system in the country. The Ministry of Education has started to work on defining new standards for the input of colleges of education, and admission to faculties of education will be subject to criteria, characteristics and skills that applicants must have to raise the level of admission to teacher preparation programs. However, the reality on the ground has not yet changed, except for stopping bachelor’s degree programs for teacher preparation at universities in the country. Improving the status of teaching could be followed by a stricter approach for selection. Having high abilities and motivation are important factors in the teacher selection. This would raise the likelihood of having more teachers who are willing to make more effort to help their students and improve their competencies.

Another implication could be the expansion of female teachers in the foundation years for all students. It is an approach in which the MOE started to experiment with a limited number of schools. In the past few years, Saudi Arabia’s Ministry of Education began to provide the opportunity for families in a small percentage of schools and private schools to have female teachers teach boys and girls until the third grade. This change may help in the near future to understand the extent of this impact on student performance.

## Limitation and future avenues

Finally, for future research, it would be informative to examine similar data from other countries in which boys evaluate female teachers and girls evaluate male teachers. Some might argue that girls might have different responding styles that inflate their teacher ratings. However, the clear correlation between these responses and students’ achievements weakens this argument. Overall, this study considered a set of variables, but there are other factors that could be important such as teacher enthusiasm (e.g., Keller et al., 2016) and adoption of motivating styles (e.g., Aelterman et al., 2019; Moè and Katz, 2022). Beside the variable that were examined in this study, future research could consider teacher enthusiasm and adoption of a motivating style as they might impact student learning (e.g., De Beni and Moè, 2003; Moè et al., 2021) and motivation (Kunter et al., 2011). In addition, the results of this study should be read in light of the students’ age stage, as the sample of this study was students in 4th grade, which might not apply to older students in middle and high schools.

## Conclusion

This study is the first to examine teachers’ characteristics on students’ achievement using TIMSS data. On the one hand, this study highlighted the impact of teachers’ practices and attitudes on students’ achievement. On the other hand, the study highlights the importance of taking gender differences into account not only at the student level but also at the teacher level. Female teachers evidently outperformed male teachers in practices and attitudes. Therefore, attitudes toward teaching should be an important factor when recruiting teachers’ procedures.

These results show us that there is an opportunity for improvement to first fill the gap between boys and girls with the improvement of boys’ scores to the girls’ level. This is an important step forward to improve students’ scores in Saudi Arabia in comparison to students from high-achieving countries.

Last, this is an exploratory study to discuss some noticeable statistical results through international tests. It is important that this study be followed by several studies that attempt to understand the discrepancy between the performance of boys and girls students by studying the variables that might be related students achievements, such as teachers, schools and the way schools are chosen for male and female students in these tests. After accumulating a set of results from different studies within the context of Saudi Arabia or a similar context, it may be appropriate to draw clear recommendations about what should be done by decision-makers in the Ministry of Education.

## Data availability statement

Publicly available datasets were analyzed in this study. This data can be found at: https://timss2019.org/international-database/.

## Ethics statement

Ethical review and approval, and written informed consent, were not required for this study in accordance with the local legislation and institutional requirements.

## Author contributions

All authors listed have made a substantial, direct, and intellectual contribution to the work and approved it for publication.

## Funding

This study is supported *via* funding from Prince Sattam bin Abdulaziz University project number (PSAU/2023/R/1444).

## Conflict of interest

The authors declare that the research was conducted in the absence of any commercial or financial relationships that could be construed as a potential conflict of interest.

## Publisher’s note

All claims expressed in this article are solely those of the authors and do not necessarily represent those of their affiliated organizations, or those of the publisher, the editors and the reviewers. Any product that may be evaluated in this article, or claim that may be made by its manufacturer, is not guaranteed or endorsed by the publisher.

## Author disclaimer

All claims expressed in this article are solely those of the authors and do not necessarily represent those of their affiliated organizations, or TIMSS & PIRLS International Study Center.
